# Parenteral, Non-Intravenous Analgesia in Acute Traumatic Pain—A Narrative Review Based on a Systematic Literature Search

**DOI:** 10.3390/jcm13092560

**Published:** 2024-04-26

**Authors:** Midas N. de Grunt, Bianca de Jong, Markus W. Hollmann, Milan L. Ridderikhof, Robert P. Weenink

**Affiliations:** 1 Department of Anaesthesiology, Amsterdam UMC, 1105 AZ Amsterdam, The Netherlands; m.n.degrunt@amsterdamumc.nl (M.N.d.G.); biancadejong1298@gmail.com (B.d.J.); m.w.hollmann@amsterdamumc.nl (M.W.H.); 2 Department of Emergency Medicine, Amsterdam UMC, 1105 AZ Amsterdam, The Netherlands; m.l.ridderikhof@amsterdamumc.nl

**Keywords:** acute pain, trauma, wounds and injuries, analgesia, emergency medical services, emergency service, hospital, adults

## Abstract

Traumatic pain is frequently encountered in emergency care and requires immediate analgesia. Unfortunately, most trauma patients report sustained pain upon arrival at and discharge from the Emergency Department. Obtaining intravenous access to administer analgesics can be time-consuming, leading to treatment delay. This review provides an overview of analgesics with both fast onset and parenteral, non-intravenous routes of administration, and also indicates areas where more research is required.

## 1. Introduction

Traumatic pain is common in the prehospital environment and Emergency Department (ED); more than two-thirds of trauma patients report moderate to severe pain upon arrival at and discharge from the ED [1]. Inadequate analgesia hampers access to the patient, delays necessary treatments and transportation, increases patient suffering, and is frequently associated with a more profound stress response [2].

Treatment of traumatic pain often relies on intravenous (IV) access, since IV administration leads to the fastest onset [3]. However, obtaining IV access takes time and is not always easy, leading to treatment delay [4,5,6]. Other routes of administration, such as inhalation, intranasal (IN), or oromucosal administration, offer specific advantages compared to IV administration regarding ease of use and time management. Compared to oral administration, these routes offer advantages in terms of faster onset and increased biological availability due to bypassing gastro-intestinal degradation and hepatic first-pass metabolism [3]. 

One step towards improving prehospital and ED management of traumatic pain might be eliminating the need for immediate IV access to provide analgesia. Therefore, this literature review summarizes the evidence for the most relevant alternative routes of administration and commonly used analgesics for situations where rapid analgesia is required but IV access is not (readily) available. It also identifies areas where more research is required.

## 2. Methods

### 2.1. Search 

We performed a systematic literature search of Medline, Embase, and Cochrane from inception up to 6 February 2024 using the search strategy provided in Supplementary File S1. An information specialist was consulted for optimization of the search.

### 2.2. Inclusion and Exclusion Criteria 

Eligibility criteria were defined using the PICO framework. The population consisted of adults receiving emergency care for acute (<48 h), moderate to severe (Numeric Rating Scale (NRS) ≥ 4 or Visual Analogue Scale (VAS) ≥ 40 mm), traumatic pain. The intervention consisted of parenteral, non-intravenous analgesia. A comparator was not defined. The outcome was pain intensity or safety. Studies were excluded if they did not present original data; were conference abstracts, case reports, or series; measured the first endpoint beyond 30 min; were not available in full text, or were written in a language other than English or Dutch.

### 2.3. Article Screening 

Titles and abstracts were screened by two contributors (MG and BJ). Conflicting inclusions and exclusions were resolved by discussion, and a third contributor (RW) was consulted if consensus could not be reached. Following inclusion, full-text articles were screened similarly. Additional eligible articles were identified by backward snowballing.

## 3. Results

**Figure 1:**
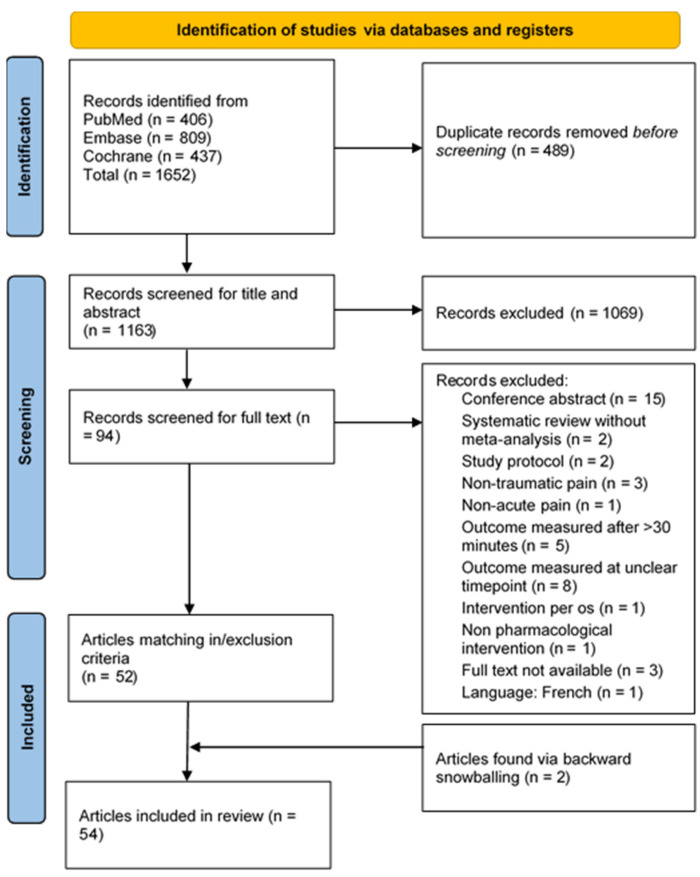
PRISMA 2020 flow diagram.

Our literature search yielded 1652 results, of which 489 duplicates were removed. After initial screening of the title and abstract, 94 articles proceeded to full-text review, which yielded 52 eligible articles. Backward snowballing provided two additional eligible articles, resulting in 54 eligible articles for this review (Figure 1). An overview of all eligible articles is provided in Supplementary File S2.

Most studies focused on either inhalation (n = 19), intranasal (n = 17), or oromucosal (n = 6) drug administration. The most frequently used analgesics were methoxyflurane (MOF, n = 12), fentanyl (n = 9), ketamine (n = 7), nitrous oxide (N_2_O, n = 6), and sufentanil (n = 6). These routes of administration and analgesics are included in this review. For each analgesic, an overview of the available data is included in Supplementary File S3. Only key data are presented in the review, using, when available, (1) meta-analyses or randomized controlled trials (RCTs), where (2) the specific analgesic is used as the primary therapy and (3) standard-of-care (SoC) analgesics are used as comparators. When these data are not available or insufficient, supportive data from other studies are presented. The clinical relevance of any reported differences is also discussed. What is regarded as the minimal clinically relevant difference in pain intensity varies greatly across studies, ranging from 8 to 40 mm (on a 100 mm scale), and also depending on the baseline pain score [7]. In this review, differences smaller than 1.0 on NRS or 10 mm on VAS are considered clinically irrelevant. For differences larger than this threshold, clinical relevance is assessed by combining pain intensity reduction with other outcomes, such as patient satisfaction and safety.

## 4. Routes of Administration

### 4.1. Inhalation

The respiratory tract is a non-invasive route of administration and potentially offers superior bioavailability to all other non-invasive routes of administration. Absorption of small (<1000 Dalton) molecules is mainly based on lipophilicity, being absorbed within a few minutes [8]. A challenge of drug administration via inhalation is obtaining optimal particle deposition. The quickest and most efficient absorption into the systemic circulation occurs through the alveolar membrane. To reach the alveoli, a particle has to pass mechanical (bifurcations, mucociliary clearance), chemical (enzymatic degradation), and immunological (phagocytosis) barriers [9]. It is also strongly dependent on correct use of the inhaler or nebulizer. This review includes inhaled MOF, N_2_O, nebulized fentanyl, and ketamine.

### 4.2. Intranasal Administration

The nasal mucosa consists of highly vascularized and permeable tissue. Advantages of IN administration include ease of use, rapid onset of action, and direct entry to the central nervous system through the olfactory region [10]. An ideal particle for IN administration has a low molecular weight (MW) and high lipophilicity, is non-ionized at physiological pH, and is dissolved in a solution with low viscosity and administered at a 30-degree angle above horizontal [11,12,13,14]. The optimal volume for IN administration is 100–150 µL per nostril, but clinical guidelines often accept volumes up to 1 mL per nostril [14,15,16,17]. This review includes IN fentanyl, ketamine, and sufentanil.

### 4.3. Oromucosal Administration

Oromucosal drug administration, primarily targeting the buccal and sublingual tissue, is painless and does not require technical equipment or expertise. The oral mucosa presents both hydrophilic and lipophilic barriers which limit drug absorption, restricting oromucosal drug administration to drugs with high potency [18]. Other disadvantages include substantial inter- and intra-individual variation in drug absorption and limited applicability in cases of nausea or vomiting [18]. This review includes fentanyl buccal tablets (FBT) and oral transmucosal fentanyl citrate (OTFC).

## 5. Analgesics

### 5.1. Methoxyflurane

MOF is an inhalational anesthetic and comes in a hand-held inhaler known as the “green whistle”. Its physicochemical properties enable quick absorption through the alveolar membrane: a low MW (165 Dalton) and high lipophilicity (oil/gas partition coefficient: 825) [19,20]. An overview of studies and key outcomes is provided in Supplementary File S3 (Table S9). Two recent meta-analyses are discussed below [21,22].

Fabbri et al. included four RCTs (STOP!, InMEDIATE, MEDITA, and PenASAP) with a total of 1090 subjects, comparing MOF to SoC and/or placebo [21]. SoC generally included paracetamol, NSAIDs, and weak opioids for moderate pain and IV opioids for severe pain. MOF led to a stronger reduction in pain intensity measured with VAS during the first 30 min (estimated treatment difference 11.9 mm (95%CI 9.8, 14.0), *p* < 0.0001). MOF also led to a stronger reduction in pain intensity in comparison with each individual treatment category (placebo, paracetamol, NSAIDS and opioids). Liu et al. included the four before mentioned RCTs, an additional RCT (RAMPED) and four subgroup analyses (two subgroup analyses of the STOP! trial and two subgroup analyses of the MEDITA trial) with a total of 1806 subjects, comparing MOF to SoC and/or placebo [22]. MOF led to a stronger reduction in pain intensity measured with NRS at 3, 5, 10, 15, and 20 min. These differences could represent clinical benefits, since other outcomes were also in favor of MOF: time to patient-reported pain relief was shorter and overall efficacy was rated higher by patients, physicians, and nurses. Adverse events were more common after the use of MOF (RR 3.09 (95%CI 1.72, 5.57), *p* = 0.0002) and included dizziness, somnolence, and feeling drunk [22]. No significant effects on vital variables were reported [23,24,25]. 

The presented meta-analyses are not without limitations. Fabbri et al. did not systematically assess methodological quality or risk of bias and did not report data on possible heterogeneity. Liu et al. reported a high risk of bias in multiple included RCTs [24,26], and the quality of evidence was (very) low for most outcomes. Liu et al. included a trial also including non-trauma patients (RAMPED [27]) and multiple analyses of the same patient cohorts of the STOP! [23,28,29] and MEDITA [24,30,31] trial, which was methodologically flawed because some patients were included multiple times. Moreover, the company that produces MOF was involved with multiple RCTs [24,25,26,28] and one meta-analysis [21], possibly leading to industry sponsorship bias. 

#### Conclusion

MOF leads to a stronger reduction in pain intensity compared to SoC and/or placebo. Patient-reported pain relief was earlier and satisfaction with overall efficacy was rated higher by patients and healthcare providers. Therefore, MOF is recommended for the management of moderate to severe traumatic pain. A disadvantage is increased cost: Smith et al. calculated that providing pain relief using MOF costs almost twice as much compared to the combination of morphine IV and paracetamol IV [32]. There might also be concerns about the environmental impact of inhalational anesthetics. However, the 100-year global warming potential of MOF is significantly lower than that of other inhalational anesthetics such as N_2_O, and the ozone depletion potential is negligible [33]. A complete life cycle assessment is currently not available.

### 5.2. Nitrous Oxide

N_2_O (“laughing gas”) is an inhalational anesthetic with both analgesic and anxiolytic effects. Its physicochemical properties ensure a very rapid onset (blood/gas partition coefficient 0.45), but result in a relatively low potency (oil/gas partition coefficient 1.4) [34]. An overview of studies and key outcomes is provided in Supplementary File S3 (Table S10). 

The analgesic efficacy of N_2_O in trauma patients is reported to be similar to SoC IV analgesics [35,36]. Kariman et al. compared N_2_O/O_2_ (50:50) to fentanyl IV (2 µg/kg) and found similar pain intensity as that measured with VAS (on a 0–10 scale) at 3, 6, and 60 min [35]. The pain intensity was lower at nine minutes (2.2 vs. 3.1, difference −0.9 (95%CI −1.7, −0.1), *p* = 0.006), but the difference was considered clinically irrelevant. Pain intensity was not recorded between 9 and 60 min. The incidence of adverse events was similarly lacking effects on vital variables. Motamed et al. compared N_2_O/O_2_ (50:50) to ketamine IV (0.3 mg/kg) and reported similar pain intensity as that measured with VAS (on a 0–10 scale) at 5, 10, and 15 min [36]. However, the pain intensity scores at 15 min seemed substantially different for N_2_O and ketamine (5.1 vs. 2.5, respectively), which was also reflected in a higher need for rescue medication at 15 min: 60% vs. 5%. Since information on the spread of data or *p*-values was not reported for various analyses, including pain intensity scores, the findings are difficult to interpret.

Supportive data are similarly limited. Ducasse et al. compared N_2_O/O_2_ (50:50) to medical air for 15 min and found a higher percentage of patients experiencing pain relief (defined as NRS ≤ 3) at 15 min following N_2_O/O_2_: 67% vs. 27%, difference = 40% (95%CI 17, 63), *p* < 0.001 [37]. Adverse events were not reported up to this time. Porter et al. set out to perform a meta-analysis assessing the analgesic effectiveness of N_2_O/O_2_ and MOF [38]. Strikingly, the authors could not identify any head-to-head trials making indirect treatment comparison (using placebo as a bridging comparator) necessary. Changes in pain intensity were similar at 5, 10, and 15 min. A safety assessment was not possible.

#### Conclusion

High-quality trials in adult trauma patients comparing N_2_O to SoC analgesics are lacking. It should also be considered that N_2_O is contra-indicated in a variety of traumatic conditions, for example, in the possible presence of air cavities such as after traumatic brain injury (pneumocephalus) or thoracic trauma (pneumothorax). To conclude, the use of N_2_O for the management of traumatic pain cannot be recommended. Considering the recent registration of MOF, which offers increased portability and lower environmental impact, it is not likely that RCTs assessing efficacy of N_2_O in traumatic pain will be initiated in the future.

### 5.3. Fentanyl

Fentanyl is a small (MW 335 Dalton) and highly lipophilic drug (n-octanol/water partition coefficient 860), enabling IN and oromucosal administration, as well as inhalation [39]. It is included in many international acute pain management guidelines, mainly describing IV and IN administration [15,16,40,41,42]. Oromucosal administration is included in prehospital military guidelines [42]. An overview of studies and key outcomes is provided in Supplementary File S3 (Table S11). 

#### 5.3.1. Intranasal Fentanyl

Interestingly, no head-to-head comparisons between fentanyl IN and current SoC IV analgesics were identified. Isfahani et al. compared fentanyl IN (1 µg/kg) to ketamine IN (1 mg/kg) and placebo IN (all combined with paracetamol) [43]. Pain intensity, as measured with VAS, was similar for fentanyl IN and placebo IN at 5, 10, and 30 min. IN medication was well tolerated, but patient satisfaction was low for all groups. Although the dose used for fentanyl IN was relatively low (1 µg/kg), the results were both disappointing and surprising, considering that fentanyl IN is included in numerous pain management guidelines. A retrospective registry study of fentanyl IN (1–2 µg/kg) for prehospital analgesia by ski patrols found significant pain reduction after five minutes without adverse events [44]. The conclusions from these studies are even more limited due to methodological flaws: the study by Isfahani et al. was underpowered, lacked an IV comparator, and excluded patients experiencing AEs or lack of pain control.

Fentanyl IN has also been studied as a supplemental analgesic: Chew et al. used fentanyl IN (1.5 µg/kg) on top of background treatment with tramadol IV (2 mg/kg) and compared it to monotherapy with tramadol IV (2 mg/kg) [45]. Fentanyl IN + tramadol IV led to a stronger reduction in pain intensity as measured with VAS at ten minutes (29.8 mm vs. 19.6 mm, difference: 10.2 mm (95%CI 1.7, 18.8), *p* = 0.022). The clinical relevance of this difference is questionable, since no subjects in either group required additional analgesia. It should be noted that fentanyl IN was administered during the five minutes before the administration of tramadol. Consequently, fentanyl IN was given more time to obtain its effect. Moreover, the combination of IV and IN analgesics does not seem very realistic, because IN administration is usually intended for situations where IV access is not readily available. 

#### 5.3.2. Fentanyl Buccal Tablets

Shear et al. and Arthur et al. both compared FBT to oxycodone/paracetamol tablets [46,47]. Shear et al. used the lowest available dose for FBT (100 µg) and compared it to a similarly low dose of oxycodone/paracetamol tablets (5/325 mg) [46]. FBT led to a shorter time to significant pain relief (>2 points NRS reduction): 10 min (IQR 5, 15) vs. 35 min (IQR 2, 40), *p* = 0.0001. The time to maximal pain reduction was shorter, and fewer patients required rescue medication. A follow-up study by Arthur et al. used an increased dose of FBT (200 µg) and oxycodone/paracetamol tablets (10/325 mg), which, according to the authors, better reflected SoC dosages [47]. They found no differences in any pain relief endpoints, but since the sample was small (n = 50) and a sample size calculation was not performed, the study might have been underpowered. 

#### 5.3.3. Oral Transmucosal Fentanyl Citrate

OTFC incorporates fentanyl citrate into lozenges and is registered for the treatment of malignant breakthrough pain, but is also used in Tactical Combat Casualty Care [42]. A retrospective registry study from the prehospital battlefield environment found that OTFC provided significant pain relief within 30 min, and only 18% of patients required additional analgesia [48]. Most patients received a dose of 800 µg or higher. Adverse effects were minor, with only one major adverse side effect of hypoventilation in a patient that received 3200 µg OTFC and 20 mg morphine IV. 

#### 5.3.4. Nebulized Fentanyl

Farahmand et al. compared nebulized fentanyl (4 µg/kg) to morphine IV (0.1 mg/kg) and found a similar reduction in pain intensity as measured with NRS at ten minutes [49]. The reduction in pain intensity was stronger at 30 min (5.0 (95%CI 4.7, 5.2) vs. 4.5 (95%CI 4.3, 4.8), *p* = 0.006). The difference is considered clinically irrelevant, which is also reflected by a similar proportion of patients requiring rescue medication and similar patient satisfaction. Adverse events were more common for morphine. Maleki Verki et al. compared nebulized fentanyl (4 µg/kg) to ketamine IV (0.4 mg/kg) and found higher pain intensity as measured with NRS in the fentanyl group at 10 min (5.6 vs. 4.8, *p* = 0.001) and 30 min (3.7 vs. 2.1, *p* = 0.001) [50]. The difference at 30 min could represent a clinically relevant difference. 

#### 5.3.5. Conclusion

Although fentanyl can be administered through several routes, studies in adult trauma patients are very limited and not of high quality. Some other factors also need to be considered. First, the use of fentanyl for the treatment of traumatic pain is off-label. Second, fentanyl has been associated with misuse, abuse, and addiction, especially in North America [51]. Third, opioids are associated with opioid-induced hyperalgesia, although it should be mentioned that the current evidence for fentanyl-induced hyperalgesia after short-term use in acute pain is conflicting [52,53,54]. To conclude, the use of fentanyl through these routes of administration for the management of traumatic pain cannot be recommended, and more research is required. 

### 5.4. Ketamine

Ketamine, administered IV or IN, is often used as alternative for opioids in civilian and military acute pain management guidelines [15,40,41,42]. Some European countries use esketamine, the S-enantiomer of racemic ketamine, which has two times the analgesic potency of racemic ketamine [55,56]. The reported bioavailability for IN (es)ketamine ranges from 30–50%, significantly lower than that of fentanyl [57,58,59]. An overview of the studies and key outcomes regarding ketamine is included in Supplementary File S3 (Table S12). Studies on esketamine were not found.

#### 5.4.1. Intranasal Ketamine

Two studies compared ketamine IN to current SoC IV analgesics. Shimonovich et al. compared ketamine IN (1 mg/kg) to morphine IV (0.1 mg/kg) and morphine IM (0.15 mg/kg) and found a similar time to clinically relevant pain reduction (≥15 mm VAS reduction) for ketamine IN and morphine IV: 14.3 min (95%CI 9.8, 18.8) vs. 8.9 min (95%CI 6.6, 11.2), *p* = 0.300 [60]. However, the difference seems substantial, and one might argue for a type II error, since it is unclear whether the study was appropriately powered. The maximum pain reduction and patient satisfaction were also similar. Parvizrad et al. compared ketamine IN (0.4 mg/kg) to ketamine IV (0.2 mg/kg) and found a similar change in pain intensity as that measured with VAS at 30 min [61]. However, more patients in the IN group required an additional dose of the study medication after ten minutes because they did not experience clinically relevant pain reduction (VAS reduction > 30 mm): 63.6% vs. 0%.

Supportive data from observational studies or studies using ketamine IN as a supplemental analgesic show high proportions of patients with adequate pain relief, high patient satisfaction, and less of a need for supplemental analgesia during their ED stays [62,63,64]. Ketamine IN is well tolerated and safe, but is associated with a high incidence of mild and transient AEs (incidence ranging from 18 to >88%), including dizziness, fatigue, and nausea and vomiting [60,61,62,63,64].

#### 5.4.2. Nebulized Ketamine

Aramugam et al. compared nebulized ketamine (50 mg) to N_2_O/O_2_ (50:50) and found a similar reduction in pain intensity to that measured with VAS (on a 0–10 scale) at 5 and 30 min [65]. No patients required rescue analgesia, and patient satisfaction was similar. The inability to detect a difference might be due to insufficient power, since the required sample size was not reached.

#### 5.4.3. Conclusion

Ketamine IN may provide analgesia that is similar to other SoC IV analgesics, but high-quality RCTs are required. Ketamine IN as a supplemental analgesic reduces the need for supplemental analgesia with opioids during a patient’s ED stay. Evidence on the efficacy and safety of nebulized ketamine cannot be assessed.

### 5.5. Sufentanil

Sufentanil is a synthetic µ-opioid receptor agonist with a potency 7–10 times higher than that of fentanyl [66]. Sufentanil is highly suitable for IN administration and sublingual administration: it is more lipophilic than fentanyl, has a low MW (387D, similar to fentanyl), and has a bioavailability of 78% when administered IN as drops (i.e., non-nebulized) [66,67,68]. An overview of studies and key outcomes is presented in Supplementary File S3 (Table S13).

#### 5.5.1. Intranasal Sufentanil

Blancher et al. performed a non-inferiority trial comparing sufentanil IN (0.30 µg/kg) to morphine IV (0.1 mg/kg) [69]. The primary outcome was a reduction in pain intensity, as measured with NRS at 30 min, and the non-inferiority threshold was set at −1.3. Sufentanil IN was non-inferior and superior compared to morphine IV (−5.2 vs. −4.1, mean difference 1.1 (97.5%CI 0.3, 1.9), *p* < 0.001). NRS reduction at 10 and 20 min was similar, as was the incidence of adverse events. The clinical relevance of the difference found at 30 min is debatable, since patient satisfaction was similar. 

Sufentanil IN has also been used in multimodal analgesia protocols. Malinverni et al. compared two protocols consisting of paracetamol, an NSAID, and an opioid [70]. The intervention protocol included sufentanil IN (0.5 µg/kg), and the SoC protocol used oxycodone PO (5 mg) or titrated morphine IV. The intervention protocol led to a stronger reduction in pain intensity as measured with NRS at 15–20 min: 3.0 (IQR 1.7, 5.0) vs. 1.5 (IQR 0.9, 3.0), *p* < 0.001. It should be noted that only 14% of patients undergoing the SoC protocol received titrated morphine IV despite high baseline pain intensity. The incidence of adverse events was high following the intervention protocol (71.1% vs. 23%, *p* < 0.001, respectively). Lemoel et al. compared sufentanil IN (0.4 µg/kg) to placebo IN after administration in the ED triage zone [71]. All patients received additional SoC analgesics (paracetamol IV, ketoprofen IV, and titrated morphine IV) after being installed in an individual ED room, and IV access was obtained. Sufentanil IN + SoC led to a higher proportion of patients experiencing pain relief (NRS ≤ 3) at 30 min: 72.2% vs. 51.4%, a difference of 20.8 (95%CI 4.0, 36.2), *p* = 0.01. Fewer patients required titrated morphine IV following administration of sufentanil IN in the ED triage zone. However, patient satisfaction at discharge was similar, which might partially be attributed to the high incidence of AEs in the group that received sufentanil IN: 66.7% vs. 22.5%, difference 44.1% (95%CI 27.2, 57.7).

#### 5.5.2. Sublingual Sufentanil Tablets

Miner et al. assessed the feasibility of a sublingual sufentanil tablet (30 µg) [67]. The mean NRS was 8.1 at baseline, 7.0 after 15 min, and 6.2 at 30 min. Only 7.5% of patients requested rescue medication, and the ease of administration was highly rated by healthcare providers. The side effects were those traditionally related to opioids (nausea, vomiting, somnolence), and one SAE occurred: angina pectoris of moderate severity. Limitations included a lack of a comparator and a limited sample size (n = 76).

#### 5.5.3. Conclusion

Sufentanil IN provides pain relief similar to (IV) opioids, but is associated with a high incidence of (minor) adverse events. It could therefore be considered for the management of moderate to severe traumatic pain. However, the issues regarding fentanyl largely also apply to sufentanil: Its use in management of traumatic pain is off-label and there might be a risk of abuse, addiction, and opioid-induced hyperalgesia. More research on sublingual sufentanil tablets is warranted.

## 6. Discussion

This review focuses on methods of providing analgesia to adults suffering from acute, moderate to severe traumatic pain in situations where IV access is not (readily) available. Inhalation, intranasal, and oromucosal administration are available. Based on the reported studies and associated (dis)advantages, MOF is most strongly recommended. High satisfaction rates concerning ease of use and portability also make it suitable for a prehospital setting. Ketamine IN and sufentanil IN can be considered based on limited data. Although the use of nitrous oxide and fentanyl IN is common in acute pain management, the number and quality of studies on these analgesics in this population are insufficient to recommend their use. The lack of high-quality data in this population might mean that their use is based on studies in other populations (such as patients suffering from visceral pain) or due to the fact that their use has been widely accepted. There are insufficient studies in this population to assess the efficacy and safety of opioids or ketamine through inhalation.

Having considered the literature, there are several areas for future research. Most importantly, more research is required in order to appropriately determine the efficacy of fentanyl IN, ketamine IN, and sufentanil IN in traumatic pain, especially in a prehospital environment. Similarly, oromucosal administration of fentanyl and sufentanil requires more research. Ideally, these interventions would be compared to IV and SoC analgesics in double-blind RCTs. Pharmacokinetic studies for analgesics following IN and oromucosal administration could also be very insightful, since there are concerns about inter- and intra-individual variability in absorption rates. Lastly, future research could focus on optimizing analgesics for IN and oromucosal administration by optimizing the administered volume or adding vehicles to increase the residence time and permeation [72]. 

A few current RCTs are of Interest: the FORE-PAIN trial is a double-blind, multi-arm RCT comparing fentanyl IV to fentanyl IN, esketamine IV, and esketamine IN for the prehospital management of acute traumatic pain in adults (clinicaltrials.gov identifier NCT06051227). The DEEP trial is a single-blind RCT comparing sublingual sufentanil tablets to SoC for the treatment of traumatic pain in adults in the ED (clinicaltrials.gov identifier NCT05288348). Another double-blind RCT compares nebulized morphine to morphine IV for the treatment of traumatic pain in the ED (clinicaltrials.gov identifier NCT01123551).

## Supplementary Materials

The following supporting information can be downloaded at: https://www.mdpi.com/article/10.3390/jcm13092560/s1, File S1: Literature search; File S2: Overview of all eligible studies and routes of administration per analgesic (Tables S1–S8); File S3: Overview of studies and key outcomes for MOF (Table S9), N_2_O (Table S10), fentanyl (Table S11), ketamine (Table S12) and sufentanil (Table S13). References [73,74,75,76,77,78,79,80,81,82,83,84,85,86,87,88,89,90,91,92,93,94,95] have been cited in the Supplementary Materials.
