# Supplementary material for: Parenteral, Non-Intravenous Analgesia in Acute Traumatic Pain—A Narrative Review Based on a Systematic Literature Search
Source: J Clin Med. 2024 Apr 26;13(9):2560. doi: 10.3390/jcm13092560 (PMC11084350; doi:10.3390/jcm13092560)
Supplement: Supplementary file 1 [file jcm-13-02560-s001.zip › jcm-2948050-supplementary.pdf]

Supplementary file

Appendix S1: literature search

PUBMED

("Wounds and Injuries"[Mesh] OR "Fractures, Bone"[Mesh] OR trauma*[tiab] OR injur*[tiab] OR fracture*[tiab] OR wound*[tiab] OR musculoskeletal[tiab])

AND

("Acute Pain"[Mesh] OR "Pain"[Mesh] OR pain[tiab])

AND

("Emergency Medical Services"[Mesh] OR "Emergencies"[Mesh] OR "Emergency Treatment"[Mesh] OR "Emergency Medicine"[Mesh] OR emergenc*[tiab] OR prehospital[tiab] OR pre-hospital[tiab] OR ambulanc*[tiab] or out-of-hospital[tiab] or "out of hospital"[tiab])

AND

("Administration, Inhalation"[Mesh] OR "Administration, Sublingual"[Mesh] OR "Administration, Topical"[Mesh] OR "Infusions, Intra-Arterial"[Mesh] OR "Infusions, Intraosseous"[Mesh] OR "Infusions, Subcutaneous"[Mesh] OR "Injections, Intra-Arterial"[Mesh] OR "Injections, Intramuscular"[Mesh] OR "Injections, Intraocular"[Mesh] OR "Injections, Subcutaneous"[Mesh] OR "Nitrous Oxide"[Mesh] OR Inhal*[tiab] OR sublingual*[tiab] OR topical[tiab] OR buccal[tiab] OR oromucosal[tiab] OR cutaneous[tiab] OR patch[tiab] OR skin[tiab] OR mucosal[tiab] OR nasal*[tiab] OR intranasal[tiab] OR rectal[tiab] OR ophthalmic[tiab] OR intraocular[tiab])

AND

(trial*[tiab] OR random*[tiab] OR meta*[tiab] OR study[tiab])

NOT

("Child"[Mesh] OR "Rodentia"[Mesh] OR "Artiodactyla"[Mesh] OR "Lagomorpha"[Mesh])

EMBASE (OVID):

Database(s): Embase Classic+Embase 1947 to 2024 February 06
Search Strategy:

#	Searches
1	injury/ or wound/
2	bone injury/ or fracture/
3	(trauma* or injur* or fracture* or wound* or musculoskeletal).ti,ab,kf.
4	1 or 2 or 3
5	pain/
6	pain.ti,ab,kf.
7	5 or 6
8	exp emergency health service/
9	emergency/

10	emergency treatment/
11	emergency medicine/
12	(emergenc* or prehospital or pre-hospital or ambulanc* or "out of hospital").ti,ab,kf.
13	8 or 9 or 10 or 11 or 12
14	inhalational drug administration/
15	sublingual drug administration/
16	topical drug administration/
17	intraarterial drug administration/
18	intraosseous drug administration/
19	subcutaneous drug administration/
20	intraarterial drug administration/
21	intramuscular drug administration/
22	intraocular drug administration/
23	subcutaneous drug administration/
24	nitrous oxide/
25	(Inhal* or sublingual* or topical or buccal or oromucosal or cutaneous or patch or skin or mucosal or nasal* or intranasal or rectal or ophthalmic or intraocular).ti,ab,kf.
26	14 or 15 or 16 or 17 or 18 or 19 or 20 or 21 or 22 or 23 or 24 or 25
27	(trial* or random* or meta* or study).ti,ab,kf.
28	4 and 7 and 13 and 26 and 27
29	child/ or exp rodent/ or exp Artiodactyla/ or exp lagomorph/
30	28 not 29

Cochrane Central Register of Controlled Trials

Issue 2 of 12, February 2024

ID Search Hits
#1 (trauma* or injur* or fracture* or wound* or musculoskeletal):ti,ab,kw 154299
#2 (emergenc* or prehospital or pre-hospital or ambulanc* or "out of hospital"):ti,ab,kw 41141
#3 (pain):ti,ab,kw 227788
#4 (Inhal* or sublingual* or topical or buccal or oromucosal or cutaneous or patch or skin or mucosal or nasal* or intranasal or rectal or ophthalmic or intraocular):ti,ab,kw 209779
#5 #1 AND #2 AND #3 AND #4 in Trials 567
#6 (child OR rodentia OR artiodactyla OR lagomorpha):ti,ab,kw 176483
#7 #5 not #6 416

Appendix S2 - overview of all eligible articles per analgesic

Table S1: Overview of all studies on methoxyflurane in adult trauma patients

AUTHOR + YEAR	STUDY DESIGN	SETTING	INTERVENTION	COMPARATOR
**FABBRI ET AL, 2021 (21)**	Meta-analysis	ED/EMS	MOF	SoC and/or placebo
**LIU ET AL, 2021 (22)**	Meta-analysis	ED/EMS	MOF	SoC and/or placebo
**BOROBIA ET AL, 2020 (26)**	Open-label multicenter RCT	ED	MOF	SoC
**COFFEY ET AL, 2014 (28)**<br><br>**SUBGROUP ANALYSIS:**<br>**COFFEY ET AL, 2016 (23)**	Double-blind multicenter RCT	ED	MOF	Placebo
**MERCADANTE ET AL, 2019 (24)**<br><br>**SUBGROUP ANALYSES:**<br>**SERRA ET AL, 2020 (30)**<br>**VOZA ET AL, 2020 (31)**	Open-label multicenter RCT	ED	MOF	SoC

**RICARD-HIBON ET AL, 2020 (25)**	Double-blind multicenter RCT	ED	MOF + SoC	Placebo + SoC
**WONG ET AL, 2022 (73)**	Open-label single center non-inferiority RCT	ED	MOF	Ketorolac IM 30 mg
**LIM ET AL, 2021 (74)**	Phased, cluster-randomized crossover trial	EMS	MOF	Tramadol IM 50 mg
**EGGER ET AL, 2023 (75)**	Observational study	EMS	MOF	-
**TRIMMEL ET AL, 2022 (76)**	Observational study	EMS	MOF	-
**RYDLÖV ET AL, 2023 (77)**	Quality assessment study	Ski patrol	MOF	-
**PORTER ET AL, 2018 (38)**	Systematic review with indirect treatment comparison	ED / EMS	MOF	N_2O/O_2

ED = emergency department, EMS = emergency medical services, IM = intramuscular, MOF = methoxyflurane, N_2O/O_2 = nitrous oxide / oxygen mixture, RCT = randomized controlled trial, SoC = standard of care.

Table S2: overview of all eligible studies on nitrous oxide in adult trauma patients

AUTHOR + YEAR	STUDY DESIGN	SETTING	INTERVENTION	COMPARATOR
**KARIMAN ET AL, 2011 (35)**	Open-label single center RCT	ED	N_2O/O_2	Fentanyl IV
**MOTAMED ET AL, 2017 (36)**	Open-label single center RCT	ED	N_2O/O_2	Ketamine IV

**DUCASSE ET AL, 2013 (37)**	Double-blind multicenter RCT	EMS	N_2O/O_2	15 minutes of medical air, followed by N_2O/O_2
**GAO ET AL, 2019 (78)**	Double-blind single center RCT	ED	N_2O/O_2 + SoC	O_2 + SoC
**ARUMUGAM ET AL, 2022 (65)**	Open-label single center RCT	ED	Nebulized ketamine	N_2O/O_2
**PORTER ET AL, 2018 (38)**	Systematic review with indirect treatment comparison	ED/EMS	MOF	N_2O/O_2

ED = emergency department, EMS = emergency medical services, IV = intravenous, MOF = methoxyflurane, N_2O/O_2 = nitrous oxide / oxygen mixture, RCT = randomized controlled trial, SoC = standard of care.

Table S3: overview of all eligible studies on fentanyl in adult trauma patients

AUTHOR + YEAR	STUDY DESIGN	SETTING	INTERVENTION	COMPARATOR
**ISFAHANI ET AL, 2022 (43)**	Double-blind multicenter RCT	ED	Fentanyl IN + paracetamol IV OR ketamine IN + paracetamol IV	Placebo IN + paracetamol IV
**LYNCH ET AL, 2022 (44)**	Retrospective registry study	Ski patrol	Fentanyl IN	-
**CHEW ET AL, 2017 (45)**	Open-label single center RCT	ED	Fentanyl IN + tramadol IV	Tramadol IV
**SHEAR ET AL, 2010 (46)**	Double-blind single center RCT	ED	Fentanyl buccal tablets	Oxycodone/paracetamol tablets
**ARTHUR ET AL, 2015 (47)**	Double-blind single center RCT	ED	Fentanyl buccal tablets	Oxycodone/paracetamol tablets

**WEDMORE ET AL, 2012 (48)**	Retrospective registry study	Prehospital battlefield	Oral transmucosal fentanyl citrate	-
**FARAHMAND ET AL, 2014 (49)**	Double-blind single center RCT	ED	Nebulized fentanyl	Morphine IV
**VERKI ET AL, 2019 (50)**	Double-blind single center RCT	ED	Nebulized fentanyl	Ketamine IV
**JOKAR ET AL, 2018 (79)**	Single-blind single center RCT	ED	Fentanyl transdermal patches	Morphine IV

ED = emergency department, IN = intranasal, IV = intravenous, RCT = randomized controlled trial.

Table S4: overview of studies on ketamine in adult trauma patients

AUTHOR + YEAR	STUDY DESIGN	SETTING	INTERVENTION	COMPARATOR
**SHIMONOVICH, 2016 (60)**	Open-label single center RCT	ED	Ketamine IN	Morphine IV OR<br>morphine IM
**PARVIZRAD ET AL, 2017 (61)**	Triple-blind single center RCT	ED	Ketamine IN	Ketamine IV
**NASR ISFAHANI ET AL, 2022 (43)**	Double-blind multicenter RCT	ED	Ketamine IN + paracetamol IV OR fentanyl IN + paracetamol IV	Placebo IN + paracetamol IV
**MOHAMMADSHAHI ET AL, 2018 (62)**	Double-blind single center RCT	ED	Ketamine IN + morphine IV	Placebo IN + morphine IV
**BOUIDA ET AL, 2020 (63)**	Double-blind multicenter RCT	ED	Ketamine IN + SoC	Placebo IN + SoC

**SHRESTHA ET AL, 2016 (64)**	Observational study	ED	Ketamine IN	-
**ARUMUGAM ET AL, 2022 (65)**	Open-label single center RCT	ED	Nebulized ketamine	N_2O/O_2

ED = emergency department, IN = intranasal, IV = intravenous, N_2O/O_2 = nitrous oxide / oxygen mixture RCT = randomized controlled trial, SoC = standard of care.

Table S5: overview of studies on sufentanil in adult trauma patients

**AUTHOR + YEAR**	**STUDY DESIGN**	**SETTING**	**INTERVENTION**	**COMPARATOR**
**BLANCHER ET AL, 2019 (69)**	Double-blind multicenter RCT	ED	Sufentanil IN + placebo IV	Morphine IV + placebo IN
**MALINVERNI ET AL, 2024 (70)**	Open-label single center RCT	ED	Sufentanil IN + NSAID + paracetamol	Opioid PO/IV + NSAID + paracetamol
**LEMOEL ET AL, 2019 (71)**	Double-blind single center RCT	ED	Sufentanil IN + SoC	Placebo IN + SoC
**KREPS ET AL, 2019 (80)**	Open-label single center sequential trial	ED	Sufentanil IN + SoC excluding opiates	SoC
**STEENBLIK ET AL, 2012 (81)**	Observational study	Ski clinic	Sufentanil IN	-
**MINER ET AL, 2018 (67)**	Open-label multicenter trial	ED	Sufentanil sublingual tablet	-

ED = emergency department, IN = intranasal, IV = intravenous, RCT = randomized controlled trial, SoC = standard of care.

Table S6: overview of all eligible articles on other opioids (besides fentanyl and sufentanil) in adult trauma patients

AUTHOR + YEAR	STUDY DESIGN	SETTING	INTERVENTION	COMPARATOR
**JALILI ET AL, 2012 (82)**	Double-blind single center RCT	ED	Sublingual buprenorphine	Morphine IV
**LIM ET AL, 2021 (74)**	Phased, cluster-randomized crossover trial	EMS	MOF	Tramadol IM
**PIETSCH ET AL, 2021 (83)**	Observational study	Ski patrol	Nalbuphine IN	-
**SCOTT ET AL, 1994 (84)**	Open-label single center clinical trial	ED	Butorphanol IN	-
**WERMELING ET AL, 2010 (85)**	Open-label multicenter clinical trial	ED	Hydromorphone IN	-

ED = emergency department, EMS = emergency medical services, IM = intramuscular, IN = intranasal, IV = intravenous, MOF = methoxyflurane, RCT = randomized controlled trial.

Table S7: overview of all eligible articles on non-steroid anti-inflammatory drugs in adult trauma patients

AUTHOR + YEAR	STUDY DESIGN	SETTING	INTERVENTION	COMPARATOR
**QURESHI ET AL, 2019 (86)**	Double-blind single center RCT	ED	Diclofenac IM	Diclofenac PO
**SERINKEN ET AL, 2019 (87)**	Double-blind single center RCT	ED	Topical ketoprofen	Placebo
**TURNER ET AL, 2021 (88)**	Single-blind single center non-inferiority RCT	ED	Ketorolac IM	Ketorolac IM
**TURTURRO ET AL, 1995 (89)**	Double-blind single center RCT	ED	Ketorolac IM	Ibuprofen PO

ED = emergency department, IM = intramuscular, PO = per os, RCT = randomized controlled trial.

Table S8: overview of all other eligible articles in adult trauma patients

AUTHOR + YEAR	STUDY DESIGN	SETTING	INTERVENTION	COMPARATOR
**AKSEL ET AL, 2015 (90)**	Open-label single center RCT	ED	Topical lidocaine	Paracetamol IV OR ice application
**MOHAMMADKARIMI ET AL, 2014 (91)**	Double-blind single center RCT	ED	Lidocaine IN	Placebo
**TURGUT ET AL, 2022 (92)**	Double-blind single center RCT	ED	Topical lidocaine	Paracetamol IV OR dexketoprofen trometamol IV OR placebo

**KAFASH MOHAMMADJANI ET AL, 2022 (93)**	Double-blind single center RCT	ED	Topical sesame oil	Placebo
**KOCAK ET AL, 2019 (94)**	Open-label single center RCT	ED	Mesotherapy thiocolchicoside / lidocaine / tenoxicam	Dexketoprofen IV
**PICKERING ET AL, 2015 (95)**	Double-blind single center non-inferiority RCT	ED	Transmucous-buccal paracetamol	Paracetamol IV

ED = emergency department, IN = intranasal, IV = intravenous, RCT = randomized controlled trial.

Appendix S3 – overview of studies and key outcomes per analgesic

Table S9: Overview of studies on methoxyflurane in adult trauma patients

AUTHOR + YEAR	DESIGN	SETTING	INTERVENTION	COMPARATOR	N	PRIMARY OUTCOME	OTHER KEY OUTCOMES	ADVERSE EVENTS
**FABBRI ET AL, 2021 (21)**	Meta-analysis	ED/EMS	MOF 3 ml	SoC[1] and/or placebo	1090	Higher pain intensity difference as measured with VAS during first 30 min: estimated treatment difference 11.9 (95%CI 9.8, 14.0), *p* < 0.0001.	Shorter median time to patient-reported pain relief: 10 min vs 18 min, HR: 2.03 (95%CI 1.75, 2.36), *p* < 0.0001.<br><br>Higher proportion of patients who were (very) satisfied with treatment: 63.5% vs 49.2%.	Higher incidence of dizziness (16.7% vs 3.6%), somnolence (5.9% vs 0.9%) and feeling drunk (4.0% vs 0.5%).
**LIU ET AL, 2021 (22)**	Meta-analysis	ED/EMS	MOF 3 ml	SoC[1] and/or placebo	1806	Higher change in pain intensity as measured with NRS at:<br><br>3 min: WMD: − 0.4 (99% CI − 0.6, − 0.2), p < 0.00001, I^2 = 0%;<br><br>5 min: WMD: − 0.9 (99% CI − 1.1, − 0.7), p < 0.00001, I^2 = 28%;<br><br>10 min: WMD: − 1.1 (99% CI − 1.6, − 0.7), p < 0.00001, I^2 = 65%;<br><br>15 min: WMD: − 1.2 (99% CI − 2.0, − 0.5), p < 0.0001, I^2 = 85%);<br><br>20 min: WMD: − 1.1 (99% CI − 1.8, − 0.5), p < 0.00001, I^2 = 75%.	Shorter time to patient reported first pain relief: mean difference -5.29 min (95%CI -6.97, -3.62), *p* < 0.00001, I^2 = 100%.<br><br>Lower proportion of patients requiring rescue medication before discharge: RR 0.32 (95%CI 0.21, 0.49), *p* < 0.00001, I^2 = 38%.<br><br>More patients rated overall efficacy as good or higher: RR 1.31 (95%CI 1.07, 1.60), *p* = 0.009, I^2 = 86%.	More treatment-emergent AEs: RR 3.09 (95%CI 1.72, 5.57), *p* = 0.0002, I^2 = 87%.<br><br>Elevated risk of:<br><br>Dizziness: RR 4.12 (95%CI 2.69, 6.29), *p* < 0.00001, I^2 = 0%;<br><br>Somnolence: RR 3.60 (95%CI 1.84, 7.07), *p* = 0.0002, I^2 = 0%;<br><br>Feeling drunk: RR 5.43 (95%CI 2.21, 13.89), *p* = 0.0004, I^2 = 0%.

						Similar change in pain intensity as measured with NRS at:<br><br>25 min: WMD − 0.4 (99% CI − 0.9, 0.3), p = 0.06, I^2 = 3%;<br><br>30 min: WMD − 0.4; 99% CI − 1.0, 0.2; p = 0.08; I^2 = 0%.	Practicality of using MOF was rated as good or higher by more physicians (RR 1.50 (95%CI 1.29, 1.74), *p* < 0.00001, I^2 = 58%) and nurses (RR 1.89 (95%CI 1.37, 2.62), *p* = 0.0001, I^2 = 80%).	
**BOROBIA ET AL, 2020 (26)**	Open-label multicenter RCT	ED	MOF 3 ml	SoC[1]	305	Stronger mean reduction in pain intensity as measured with NRS during first 20 min: 2.5 vs 1.4, difference: 1.0 (95%CI 0.8, 1.3).<br><br>Shorter median time to first pain relief: 3.17 min (IQR 1.83, 7.44) vs 10.00 min (IQR 5.74, 14.64).	Proportion of patients requiring rescue medication was 8.5% for MOF and 12.1% for SoC.<br><br>Median patient satisfaction with pain control as measured with NRS (0 = not at all satisfied, 10 = completely satisfied) was 9.0 (IQR 8.0, 10.0) for MOF and 7.8 (IQR 6.0, 9.0) for SoC.	Incidence of AEs was 24.4% for MOF and 5.4% for SoC. Most common for MOF were dizziness (14.1%), somnolence (3.3%) and nausea (2.6%). No treatment related SAEs.<br><br>Patient and clinician satisfaction for safety as measured with NRS (0 = not at all satisfied, 10 = completely satisfied) was 9.0 (IQR 8.0, 10.0) for MOF and 9.0 (IQR 7.0, 10.0) for SoC.
**COFFEY ET AL, 2016 (23)**	Double-blind multicenter RCT	ED	MOF 3 ml	Placebo	204	Stronger mean reduction in pain intensity as measured with VAS during first 20 min: -29.0 mm vs -11.6 mm, estimated treatment effect: -17.4 mm (95%CI -22.3, -12.5), *p* < 0.0001.	Median time to first pain relief was 5.0 min (IQR 2.0, 10.0) for MOF and 20.0 min (IQR 5.0, not calculable) for placebo.	Incidence of treatment-related AEs was 42.2% for MOF and 14.9% for placebo. Most common AEs for MOF were dizziness (36.3%) and headache (19.6%). No severe adverse events or treatment related SAEs.

							Lower need for rescue medication in the first 20 min: 2.0% vs 22.8%,OR: 0.07 (95%CI 0.02, 0.29), *p* = 0.0003.<br><br>General Medication Performance (GMP) ratings by patients, physicians and research nurses were higher for MOF than placebo, *p* < 0.0001.	No observable effects on respiratory or hemodynamic variables.
**MERCADANTE ET AL, 2019 (24)[2]**	Open-label multicenter non-inferiority RCT	ED/EMS	MOF 3 ml	SoC[1]	272	Superior mean reduction in pain intensity as measured with VAS during first 10 min: -14.7 mm vs -8.8 mm, adjusted mean treatment difference: -5.9 (95%CI -8.8, -3.1), *p* <0.001.<br><br>Superior mean reduction in pain intensity as measured with VAS during first 10 min in patients with moderate pain (NRS 4-6):<br><br>-15.1 mm vs -9.2 mm, adjusted mean treatment difference: -6.0 (95%CI -9.6, -2.4), *p* = 0.001.	Superior mean reduction in pain intensity as measured with VAS at<br><br>15 min (treatment difference -5.3 mm (95% CI -9.7, -0.8), *p* = 0.020), 20 min (treatment difference -5.9 mm (95%CI -10.6, -1.2), *p* = 0.015) and 25 min (treatment difference -5.0 mm (95%CI -10.0, -0.1), *p* = 0.046), and non-inferior at 30 min (treatment difference -5.0 mm (95%CI -10.1, 0.1), *p* = 0.056).<br><br>Shorter median time to patient reported onset of pain relief: 9 min (95%CI 7.72, 10.28) vs 15 min (95%CI 14.17, 15.83).<br><br>Similar need for rescue medication: 2.2% vs 3.7%, *p* = 0.722.	Incidence of treatment-related AEs was 12.6% for MOF and 1.5% for SoC. Most common for MOF were euphoria (3.7%) and somnolence (3.0%). No SAEs.<br><br>No clinically notable changes in vital variables.

							More patients rated efficacy of treatment as good or higher: 72.7% vs 60.9%, *p* = 0.001.<br><br>More healthcare providers rated the practicality of using study treatment as good or higher: 90.3% vs 64.4%, *p* < 0.001.	
**RICARD-HIBON ET AL, 2020 (25)**	Double-blind multicenter RCT	ED	MOF 3 ml + SoC[1]	Placebo + SoC	359	Shorter median time until pain relief (VAS ≤30 mm): 35 min vs not reached, HR: 1.93 (95%CI 1.43, 2.60), *p* < 0.001.	Shorter median time to patient-reported total pain relief: 54 min vs 126 min, HR: 2.2 (95%CI 1.6, 3.1), *p* < 0.001.<br><br>Higher overall mean pain intensity difference as measured with VAS: 26.5 mm vs 17.2 mm, estimated treatment effect: 9.2 (95%CI 5.3, 13.1), *p* < 0.0001.<br><br>GMP was rated as good or higher by more patients (79% vs 54%, *p* < 0.001), nurses (69% vs 40%, *p* < 0.001) and physicians (69% vs 43%, *p* < 0.001).	Incidence of treatment related AEs was 49.2% for MOF and 12.1% for placebo. Most common for MOF were dizziness (17.9%), feeling drunk (13.4%) and somnolence (10.6%). One treatment related SAE was recorded: transient loss of consciousness, which resolved after discontinuation of MOF.<br><br>No clinically significant effects on vital variables.
**WONG ET AL, 2022 (73)**	Open-label single center	ED	MOF 3 ml	SoC: Ketorolac IM 30 mg	40	Non-inferior mean reduction in pain intensity as measured with VAS at:	Similar proportion of patients rated satisfaction with pain control as	Higher incidence of treatment-related AEs: 35% vs 0%, *p* = 0.008. AEs included dizziness,

	non-inferiority RCT					5 min: -13.9 mm vs -4.9 mm, estimated treatment effect -9.0 mm (95%CI -17.7, -0.4), *p* = 0.041;<br><br>15 min: --17.1 mm vs -14.8 mm, estimated treatment effect<br><br>-2.3 mm (95%CI -12.1, 7.6), *p* = 0.648;<br><br>30 min: -22.7 mm vs -24.0 mm, estimated treatment effect 1.3 mm (95%CI -9.5, 12.1), *p* = 0.806;<br><br>Inconclusive mean reduction in pain intensity as measured with VAS at 60 min: -30.4 mm vs -32.0 mm, estimated treatment effect: 1.6 mm (95%CI -12.7, 15.8), *p* = 0.825.	satisfied or higher: 60% vs 55%, *p* = 0.665.	drowsiness and coughing. No SAEs.<br><br>Similar changes in vital variables.
**LIM ET AL, 2021 (74)**	Phased, cluster-randomized crossover trial	EMS	MOF 3 ml	Tramadol IM 50 mg	369	Stronger median reduction in pain intensity as measured with NRS at:<br><br>5 min: 2.0 (IQR 1.0, 3.0) vs 1.0 (IQR 0.0, 2.0), *p* = 0.001;<br><br>10 min: 3.0 (IQR 1.3, 4.8) vs 1.0 (IQR 0.0, 2.0), *p* = 0.001;<br><br>15 min: 3.0 (IQR 1.5, 5.0) vs 1.0 (0.0, 2.0), *p* = 0.001;<br><br>20 min: 4.0 (IQR 1.5, 5.0) vs 1.0 (IQR 0.0, 3.3), *p* = 0.028.	Higher median patient satisfaction with effectiveness as rated on 5-point Likert scale (1-5, 1 = very dissatisfied, 5 = very satisfied): 4.0 (IQR 3.0, 5.0) vs 3.0 (3.0, 4.0), *p* <0.001.<br><br>Higher median paramedic satisfaction as rated on 5-point Likert scale regarding:	Higher incidence of AEs: 44.3% vs 6.3%, *p* < 0.001. More common were drowsiness (31.7% vs 2.8%, *p* < 0.001) and headache (4.8% vs 0.6%, *p* = 0.014).

						Shorter median time from arrival at scene to administration of treatment: 9.0 min (IQR 6.0, 14.0) vs 11.0 min (IQR 8.0, 15.0), *p* < 0.001.<br><br>Time until onset of effective analgesia (≥3 point reduction on NRS): not reported.	Ease of administration: 4.5 (IQR 4.0, 5.0) vs 4.0 (IQR 4.0, 5.0), *p* = 0.002;<br><br>Speed of onset: 4.0 (IQR 3.0, 5.0) vs 3.0 (IQR 2.0, 4.0), *p* < 0.001;<br><br>Improvement of operating conditions: 4.0 (IQR 4.0, 5.0) vs 4.0 (3.0, 4.0), *p* < 0.001.<br><br>Higher median patient satisfaction regarding effectiveness of pain relief as rated on 5-point Likert scale: 4.0 (IQR 3.0, 5.0) vs 3.0 (IQR 3.0, 4.0), *p* < 0.001.	
**EGGER ET AL, 2023 (75)**	Observational study	EMS	MOF 3 ml	-	20	Mean reduction in pain intensity as measured with NRS at 15 min: 2.9 (95% CI 2.2, 3.6), p < 0.001.	35% of subjects required additional analgesia by physician.<br><br>Mean patient satisfaction on 5 point Likert scale (1 = very satisfying, 5 = not satisfying) was 1.9 (SD 0.7).<br><br>Mean EMS clinician satisfaction on 5 point Likert scale (1 = very satisfying, 5 = not satisfying) was 1.6 (SD 0.7).	Incidence of dizziness and light-headedness was 40% and 20%, respectively.<br><br>No significant effects on vital variables.

**TRIMMEL ET AL, 2022 (76)**	Observational study	EMS	MOF 3 ml	-	109	One dose provided sufficient analgesia in 61.5% of patients.	Median time to onset of analgesia was 3.0 min (IQR 3.0, 5.0).<br><br>User satisfaction as rated by EMS personnel on 5 point Likert scale (1-5, 1 = very good, 5 = bad) was 2.0 (IQR 1.0, 3.0).	Incidence of side effects was 53.2%. Most common were dizziness (21.1%), confusion (9.2%) and feeling drunk (8.3%).<br><br>No clinically significant effects on vital variables.
**RYDLÖV, 2023 (77)**	Quality assessment study	Ski patrol	MOF 3 ml	-	53	Median reduction in pain intensity as measured with NRS at 5-10 min: 3 (IQR 2, 5), *p* < 0.001.	Effect was rated as "good", "moderate" and "no effect" by 80%, 18% and 2% of patients, respectively.	Incidence of dizziness and drowsiness was 11% and 2%, respectively.
**PORTER ET AL, 2018 (38)**	Systematic review with indirect treatment comparison	ED / EMS	MOF 3 ml	N_2O/O_2 50:50	263	Similar reduction in pain intensity at:<br><br>5 min: standardized median difference (SMD) -0.15 (95%CI<br><br>-0.76, 0.46), *p* = 0.75;<br><br>10 min: SMD -0.26 (95%CI -0.88, 0.35), *p* = 0.594;<br><br>15 min: SMD -0.20 (95%CI -0.84, 0.43), *p* = 0.688.	-	No comparison possible.

95%CI = 95% Confidence Interval, AE = adverse event, EMS = Emergency Medical Services, ED = emergency department, GMP = general medication performance, HR = hazard ratio, IM = intramuscular, ml = milliliters, MOF = methoxyflurane, min = minutes, mm = millimeter, N_2O / O_2 = nitrous oxide / oxygen mixture, NRS = numeric rating scale, OR = odds ratio, RR = risk ratio, RCT = randomized controlled trial, SAE = serious adverse event, SD = standard deviation, SMD = standardized median difference, SoC = standard of care, VAS = visual analogue scale, WMD = weighted mean difference.

[1]SoC generally included paracetamol, NSAIDs and weak opioids for moderate pain and IV opioids for severe pain.

[2]Two subgroup analyses of this trial are not presented in this table (30, 31).

Table S10: overview of studies on nitrous oxide in adult trauma patients

AUTHOR + YEAR	DESIGN	SETTING	INTERVENTION	COMPARATOR	N	PRIMARY OUTCOME	OTHER KEY OUTCOMES	ADVERSE EVENTS
**KARIMAN ET AL, 2011 (35)**	Open-label single center RCT	ED	N_2O/O_2 50:50	Fentanyl IV 2 µg/kg	100	Similar mean pain intensity as measured with VAS (0-10) at:<br><br>3 min: 5.7 vs 6.2, difference -0.5 (95%CI -1.3, 0.3), *p* = 0.089;<br><br>6 min: 3.9 vs 4.3, difference -0.4 (95%CI -1.3, 0.4), *p* = 0.239;<br><br>60 min: 1.0 vs 1.2, difference -0.2 (95%CI -0.8, 0.3), *p* = 0.406.<br><br>Lower mean pain intensity as measured with VAS (0-10) at 9 min: 2.2 vs 3.1, difference -0.9 (95%CI -1.7, -0.1), *p* = 0.006.	-	Similar incidence of AEs: 14% vs 20%, *p* = 0.424. Most common were dizziness (8% vs 4%, *p* = 0.398) and delirium-like state (4% vs 6%, *p* = 0.323).<br><br>No significant differences in vital variables.
**MOTAMED ET AL, 2017 (36)**	Open-label single center RCT	ED	N_2O/O_2 50:50	Ketamine IV 0.3 mg/kg	85[2]	Not defined	Mean pain intensity as measured with VAS (0-10) at:<br><br>5 min: 7.4 vs 7;<br><br>10 min: 6.1 vs 5.4;<br><br>15 min: 5.1 vs 2.5.	Incidence of AEs was 11.6% for N_2O and 9.5% for ketamine.

							Percentage of patients requiring rescue medication at 20 minutes was 60% for N_2O and 5% for ketamine.	
**DUCASSE ET AL, 2013 (37)**	Double-blind multicenter RCT	EMS	N_2O/O_2 50:50	15 min of medical air, followed by N_2O/O_2 50:50	60	Higher percentage of subjects with pain relief (NRS ≤3) at 15 min: 67% vs 27%, difference = 40% (95%CI 17, 63), $p < 0.001$.	98.3% of patients and 100% of healthcare personnel were (very) satisfied with analgesia.	No AEs in the first 15 min. Incidence of adverse events at 30 min was 3% for N_2O and 10% for medical air.
**PORTER ET AL, 2018 (38)**	Systematic review with indirect treatment comparison	ED/EMS	MOF 3 ml	N_2O/O_2 50:50	263	Similar change in pain intensity at:<br><br>5 min: SMD -0.15 (95%CI-0.76, 0.46), $p = 0.75$;<br><br>10 min: SMD -0.26 (95%CI -0.88, 0.35), $p = 0.594$;<br><br>15 min: SMD -0.20 (95%CI -0.84, 0.43), $p = 0.688$.	No comparison possible.	No comparison possible.
**GAO ET AL, 2019 (78)**	Double-blind single center RCT	ED	N_2O/O_2 65:35 + SoC[1]	Oxygen + SoC[1]	60	Change in pain intensity as measured with NRS at 5 and 15 min: not reported	Lower mean pain intensity as measured with NRS at:<br><br>5 min: 3.4 (SD 1.8) vs 7.0 (SD 1.8), $p < 0.01$;<br><br>15 min: 3.0 (SD 1.9) vs 6.3 (SD 2.2), $p < 0.01$.<br><br>Higher patient satisfaction as measured on 10-point scale: 8.0 (IQR 7.0, 9.0) vs 4.0 (IQR 2.0, 6.0), $p < 0.01$.	Similar incidence of AEs: 13.3% vs 3.3%, $p = 0.35$. No severe side effects.<br><br>No significant differences in vital variables.

							Higher physician satisfaction as measured on 10-point scale: 8.5 (IQR 8.0, 9.0) vs 4.0 (IQR 3.0, 6.0), *p* <0.01.	
**ARUMUGAM ET AL, 2022 (65)**	Open-label single center RCT	ED	Nebulized ketamine 50 mg	N_2O/O_2 50:50	26	Similar mean pain intensity reduction as measured with VAS (0-10) at:<br><br>5 min: 0.6 (SD 0.8) vs 0.5 (SD 0.8), *p* = 0.62;<br><br>30 min: 2.9 (SD 1.2) vs 3.0 (SD 0.6), *p* = 0.684.	No patients required rescue analgesia.<br><br>Similar mean patient satisfaction on 6-point Likert scale: 5 (satisfied) vs 5 (satisfied), *p* = 0.718.	Incidence of dizziness was 8% for ketamine and 54% for N_2O. No SAEs.<br><br>No significant effects on hemodynamic variables.

AE = adverse event, EMS = Emergency Medical Services, ED = emergency department, IV = intravenous, min = minutes, ml = milliliters, MOF = methoxyflurane, N_2O / O_2 = nitrous oxide / oxygen mixture, NRS = numeric rating scale, RCT = randomized controlled trial, SAE = serious adverse event, SD = standard deviation, SMD = standardized median difference, SoC = standard of care, VAS = visual analogue scale.

[1]SoC included paracetamol, NSAIDs and opioids.

[2]It is reported that 75 subjects were randomized, but the N_2O and ketamine group consist of 43 and 42 (total 85) subjects respectively.

Table S11: overview of studies on fentanyl in adult trauma patients

AUTHOR + YEAR	DESIGN	SETTING	INTERVENTION	COMPARATOR	N	PRIMARY OUTCOME	OTHER KEY OUTCOMES	ADVERSE EVENTS
**ISFAHANI ET AL, 2022 (43)**	Double-blind multicenter RCT	ED	Fentanyl IN 1 µg/kg + paracetamol IV 15 mg/kg OR ketamine IN 1 mg/kg + paracetamol IV<br><br>15 mg/kg	Placebo IN + paracetamol IV<br><br>15 mg/kg	150	Similar mean pain intensity as measured with VAS for fentanyl IN and placebo IN at:<br><br>5 min: 71.6 mm (SD 22.1) vs 72.4 mm (SD 22.1), *p* = 0.932;<br><br>10 min: 65.0 mm (SD 22.9) vs 66.6 mm (SD 24.3), *p* = 0.794;	Lower median patient satisfaction as measured on 11-point Likert scale (0-10, 0 = no satisfaction, 10 = complete satisfaction) for fentanyl IN compared to ketamine IN: 1.5 (range 1.0, 10.0) vs 4.0 (range 1.0, 10.0), *p* = 0.045, but similar to placebo IN: 1.5 (range 1.0, 10.0) vs 4.0 (1.0, 10.0), *p* = 0.506.	Similar incidence of AEs except for mood change which occurred more often in subjects receiving ketamine IN than fentanyl IN or placebo IN: 7.5% vs 0% vs 0%, *p* = 0.038.

						30 min: 64.3 mm (SD 24.7) vs 67.8 mm (SD 27.9), *p* = 0.520.<br><br>Higher mean pain intensity for fentanyl IN compared to ketamine IN at:<br><br>5 min: 71.6 mm (SD 22.1) vs 61.5 mm (SD 20.5), *p* = 0.044;<br><br>10 min: 65.0 mm (SD 22.9) vs 55.0 mm (SD 22.0), *p* = 0.030.<br><br>Similar mean pain intensity for fentanyl IN compared to ketamine IN at:<br><br>30 min: 64.3 mm (SD 24.7) vs 57.0 mm (SD 23.6), *p* = 0.210.	Lower median level of nasal discomfort as measured on 11-point Likert scale (0-10, 0 = no unpleasant stimulation, 10 = highest unpleasant stimulation) for fentanyl IN compared to ketamine IN: 1.0 (range 1.0, 3.0) vs 2.0 (range 1.0, 9.0), *p* = 0.005, but similar to placebo IN: 1.0 (range 1.0, 3.0) vs 1.0 (range 1.0, 7.0), *p* = 0.053.	
**LYNCH ET AL, 2022 (44)**	Retrospective registry study	Ski patrol	Fentanyl IN 1-2 µg/lg	-	247	Mean reduction in pain intensity as measured with NRS was 1.8 at 5 min, 2.4 at 10 min and 2.9 at 15 min. Pain reduction was significant from baseline, *p* < 0.0001.		No adverse events.
**CHEW ET AL, 2017 (45)**	Open-label single center RCT	ED	Fentanyl IN 1.5 µg/kg + tramadol IV 2 mg/kg	Tramadol IV 2 mg/kg	20	Stronger mean reduction in pain intensity as measured with VAS at 10 min: 29.8 mm vs 19.6 mm, difference 10.2 mm (95%CI 1.7, 18.8), *p* = 0.022.	No patients required additional analgesia at 10 min.<br><br>No patients complained of nasal irritation.	Similar incidence for:<br><br>Dizziness: 40% vs 30%, *p* = 1.0<br><br>Sleepiness: 80% vs 50%, *p* = 0.350.

								Reduction in mean arterial pressure was higher for fentanyl IN than placebo IN: 13.35mmHg vs 7.65mmHg, p = 0.029.
**SHEAR ET AL, 2010 (46)**	Double-blind single center RCT	ED	Fentanyl buccal tablets 100 µg	Oxycodone / paracetamol tablets 5/325 mg	60	Lower median time to significant pain relief (NRS reduction >2): 10 min (IQR 5, 15) vs 35 min (IQR 20, 40), p = 0.0001.	Lower median time to maximal pain reduction: 40 min (IQR 30, 50) vs 55 min (IQR 40, 60), p = 0.01.<br><br>Higher median maximum pain reduction as measured with NRS: 6 (IQR 4, 7) vs 3 (IQR 2, 5), p = 0.0004.<br><br>Similar proportion of patients experienced significant pain relief: 100% vs 83%, p = 0.052<br><br>Lower proportion of subjects required rescue medication: 17% vs 57%, = 0.003	Incidence of AEs was 13.3% for fentanyl buccal tablets and 40% for oxycodone / paracetamol. Lower incidence of nausea: 0% vs 27%, p = 0.005. Similar incidence of dizziness: 13% vs 20%, p = 0.71. No SAEs.<br><br>No significant effects on vital variables.
**ARTHUR ET AL, 2015 (47)**	Double-blind single center RCT	ED	Fentanyl buccal tablets 200 µg	Oxycodone / paracetamol tablets 10/650 mg	50	Proportion of subjects with significant pain relief (NRS reduction >2) at 10 min: not reported.	Similar rate of pain reduction as determined by multivariate Cox regression: hazard ratio: not reported (95% CI 0.4, 1.5), p = 0.28.	Similar incidence of AEs: 24% vs 20%, p = 0.73.<br><br>No abnormalities in vital variables.

							Similar proportion of subjects experienced significant pain relief at 15 min: 52% vs not reported.<br><br>Similar proportion of subjects experienced significant pain relief overall: 80% vs 88%, *p* = 0.44.	
**WEDMORE ET AL, 2012 (48)**	Retrospective registry study	Pre-hospital battle-field	Oral transmucosal fentanyl citrate	-	197	Mean pain intensity as measured with NRS decreased from 8.0 (SD 1.4) to 3.2 (SD 2.1) after 15-30 min, $p < 0.0001$.	Proportion of patients requiring additional analgesia was 18.3%.	Most common AEs were nausea (12.7%), pruritus (4.1%) and drowsiness (1.0%) 10.2% of patients received anti-emetic drugs.
**FARAHMAND ET AL, 2014 (49)**	Double-blind single center RCT	ED	Nebulized fentanyl 4 µg/kg	Morphine IV 0.1 mg/kg	90	Similar mean reduction in pain intensity as measured with NRS in first 10 min: 3.6 (95%CI 3.3, 3.9) vs 3.7 (95%CI 3.4, 3.9), *p* = 0.72.<br><br>Stronger mean reduction in pain intensity as measured with NRS at:<br>30 min: 5.0 (95%CI 4.7, 5.2) vs 4.5 (95%CI 4.3, 4.8), *p* = 0.006;<br>60 min: 5.2 (95%CI 4.9, 5.4) vs 4.6 (95%CI 4.3, 4.9), $p < 0.0001$.	Similar proportion of subjects received rescue medication: 8.5% vs 7%, *p* = 1.<br><br>Similar patient satisfaction as measured on 6-point Likert scale, *p* = 0.67.	Lower incidence of adverse events: 0% vs 8%, *p* = 0.048.<br><br>No significant changes in vital variables.
**VERKI ET AL, 2019 (50)**	Double-blind single center RCT	ED	Nebulized fentanyl 4 µg/kg	Ketamine IV 0.4 mg/kg	127	Higher pain intensity as measured with VAS (0-10) at:	Higher proportion of patients required additional analgesia at 60 min: 71% vs 0%, *p* = 0.001.	Not recorded

						10 min: 5.6 (SD 2.1) vs 4.8 (SD 3.3), *p* = 0.001;<br><br>30 min: 3.7 (SD 2.8) vs 2.1 (SD 1.4), *p* = 0.001;<br><br>60 min: 3.1 (SD 1.3) vs 2.3 (SD 0.8), *p* = 0.001.		
**JOKAR ET AL, 2018 (79)**	Single-blind single center RCT	ED	Fentanyl transdermal patches	Morphine IV 0.1 mg/kg	60	Not reported	Not interpretable	Not interpretable

AE = adverse event, ED = emergency department, IV = intravenous, IN = intranasal, NRS = numeric rating scale, min = minutes, RCT = randomized controlled trial, SAE = serious adverse event, SD = standard deviation, SoC = standard of care, VAS = visual analogue scale.

Table S12: overview of studies on ketamine in adult trauma patients

AUTHOR + YEAR	DESIGN	SETTING	INTERVENTION	COMPARATOR	N	PRIMARY OUTCOME	OTHER KEY OUTCOMES	ADVERSE EVENTS
**SHIMONOVICH, 2016 (60)**	Open-label single center RCT	ED	Ketamine IN 1 mg/kg	Morphine IV 0.1 mg/kg OR morphine IM 0.15 mg/kg	90	Similar mean time to clinically meaningful pain reduction (≥15 mm VAS) for ketamine IN and morphine IV: 14.3 min (95%CI 9.8, 18.8) vs 8.9 min (95%CI 6.6, 11.2), *p* = 0.300, but higher for morphine IM: 26.0 min (95%CI 20.3, 31.7), *p* = 0.003.	Similar mean maximal pain reduction as measured with VAS for ketamine IN and morphine IV and IM: 56 mm vs 59 mm vs 48 mm, *p* = 0.300.<br><br>Similar mean patient satisfaction as measured with VAS (100 mm) for ketamine IN and morphine IV and IM: 58.7 mm (95CI 45.3, 72.1) vs 70.2 mm (95%CI 55.2, 85.2) vs 73.9 mm (95%CI 62.9, 84.9), *p* = 0.259.	Incidence of four AEs was different among groups: difficulty concentrating, dizziness and confusion were more common for ketamine IN, while dry mouth was more common for opioids.<br><br>No significant differences in respiratory or hemodynamic variables.

**PARVIZRAD ET AL, 2017 (61)**	Triple-blind single center RCT	ED	Ketamine IN 0.4 mg/kg	Ketamine IV 0.2 mg/kg	154	Similar mean reduction in pain intensity as measured with VAS at 30 min: 43.8 mm (95%CI 41.1, 46.5) vs 46.4 mm (95%CI 42.8, 50.1), *p* = 0.245.	Higher proportion of subjects required a second dose of study medication at 10 min because they did not experience clinically significant pain reduction (VAS reduction >30 mm): 63.63% vs 0%.<br><br>62.4% of patients receiving ketamine IN had full satisfaction of painlessness.	Similar incidence of AEs: 48.1% vs 38.7%, *p* = 0.458. None required intervention. Most common side effects were fatigue (15.6%), euphoria (14.3%) and nausea (7.1%).
**NASR ISFAHANI ET AL, 2022 (43)**	Double-blind multicenter RCT	ED	Ketamine IN 1 mg/kg + paracetamol IV 15 mg/kg OR fentanyl IN 1 µg/kg + paracetamol IV 15 mg/kg	Placebo IN + paracetamol IV 15 mg/kg	150	Lower mean pain intensity as measured with VAS for ketamine IN compared to placebo IN at:<br><br>5 min: 61.5 mm (SD 20.5) vs 72.4 mm (SD 22.1), *p* = 0.032;<br><br>10 min: 55.0 (SD 22.0) vs 66.6 mm (SD 24.3), *p* = 0.047.<br><br>Similar mean pain intensity as measured with VAS for ketamine IN and placebo IN at 30 min: 57.0 mm (SD 23.6) vs 67.8 mm (SD 27.9), *p* = 0.074.<br><br>Lower mean pain intensity as measured with VAS for ketamine IN compared to fentanyl IN at:	Higher median patient satisfaction as measured on 11-point Likert scale (0-10, 0 = no satisfaction, 10 = complete satisfaction) for ketamine IN compared to fentanyl IN: 4.0 (range 1.0, 10.0) vs 1.5 (range 1.0, 10.0), *p* = 0.045, and placebo IN: 4.0 (1.0, 10.0), *p* = 0.047.<br><br>Higher level of nasal discomfort as measured on 11-point Likert scale (0-10, 0 = no unpleasant stimulation, 10 = highest unpleasant stimulation) for ketamine IN compared to fentanyl IN: 2.0 (range 1.0, 9.0) vs 1.0 (range 1.0, 3.0), *p* = 0.005, but similar to placebo IN: 1.0 (range 1.0, 7.0), *p* = 0.325.	Similar incidence of AEs, except for mood change which occurred more often in subjects receiving ketamine than fentanyl or placebo: 7.5% vs 0% vs 0%, *p* = 0.038.

						5 min: 61.5 mm (SD 20.5) vs 71.6 mm (SD 22.1), *p* = 0.044;<br><br>10 min: 55.0 mm (SD 22.0) vs 65.0 mm (SD 22.9), *p* = 0.030.<br><br>Similar mean pain intensity as measured with VAS at 30 min for ketamine IN and fentanyl IN: 57.0 mm (SD 23.6) vs 64.3 mm (SD 24.8 mm), *p* = 0.210.		
**MOHAMMADSHAHI ET AL, 2018 (62)**	Double-blind single center RCT	ED	Ketamine IN 1 mg/kg + morphine IV 0.05 mg/kg	Placebo IN + morphine IV 0.05 mg/kg	80	Lower proportion of patients requested supplemental analgesia: 30.0% vs 67.5%, *p* = 0.001.<br><br>Similar mean time until request for supplemental analgesia: 60.83 min (SD 39.19) vs 37.41 min (SD 23.95), *p* = 0.059.	Similar mean reduction in pain intensity as measured with NRS at:<br><br>10 min: -1.6 (SD 1.9) vs -1.4 (SD 1.4), *p* = 0.917;<br><br>30 min: -3.4 (SD 2.2) vs -2.9 (SD 1.8), *p* = 0.315;<br><br>60 min: -5.2 (SD 1.9) vs -4.5 (SD 2.0), *p* = 0.057.	Similar incidence of AEs: 18% vs 17.5%, *p* = 0.769. Most common was vomiting (11.3%).
**BOUIDA ET AL, 2020 (63)**	Double-blind multicenter RCT	ED	Ketamine IN 50 mg + SoC[1]	Placebo IN + SoC[1]	1102	Lower proportion of patients required opioids during ED stay: 17.2% vs 26.5%, *p* < 0.001.	Lower proportion of patients required non-opioid analgesics during ED stay: 31.3% vs 39.6%, *p* = 0.003.<br><br>Higher proportion of patients was discharged from the ED with VAS <30 mm: 80% vs 68%, *p* < 0.001.	Higher incidence of adverse effects: 43.6% vs 27.6%, *p* < 0.001. Most common for ketamine IN were dizziness (20.8%) and nausea or vomiting (15.2%).

**SHRESTHA ET AL, 2016 (64)**	Observational study	ED	Ketamine IN 0.7 mg/kg	-	39	Proportion of patients achieving pain reduction of ≥20 mm VAS at 15 min was 97%.	Median reduction in pain intensity as measured with VAS was 40 mm (IQR 24, 50) at 15 min, 50 mm (IQR 40, 70) at 30 min and 58 mm (IQR 45, 70) at 60 min.<br><br>17.6% of patients required an additional dose at 15 min.<br><br>Patient satisfaction as measured on 10-point Likert scale (1-10, 1 = not satisfied, 10 = very satisfied) was 8 (IQR 7, 9).	73.5% of patients reported no nasal irritation.<br><br>Most common side effects at 30 min were dizziness (88.2%), nausea (41.2%) and sedation (50%).<br><br>No significant differences in respiratory or hemodynamic variables.
**ARUMUGAM ET AL, 2022 (65)**	Open-label single center RCT	ED	Nebulized ketamine 50 mg	N_2O/O_2 50:50	26	Similar mean reduction in pain intensity as measured with VAS (0-10) at:<br><br>5 min: 0.6 (SD 0.8) vs 0.5 (SD 0.8), *p* = 0.62;<br><br>30 min: 2.9 (SD 1.2) vs 3.0 (SD 0.6), *p* = 0.684.	No patients required rescue analgesia.<br><br>Similar mean patient satisfaction (*p* = 0.718), but values are not reported.	Incidence of dizziness was 8% for ketamine and 53% for nitrous oxide. No SAEs.<br><br>No significant effects on hemodynamic variables.

AE = adverse event, ED = emergency department, IN = intranasal, IV = intravenous, min = minutes, mm = millimeter, NRS = numeric rating scale, RCT = randomized controlled trial, SAE = serious adverse event, SD = standard deviation, SoC = standard of care, VAS = visual analogue scale.

[1]SoC included paracetamol IV and ketoprofen IV (VAS 30-50 mm), tramadol subcutaneously (VAS 51-69 mm) and morphine IV (VAS >70 mm).

Table S13: overview of studies on sufentanil in adult trauma patients

AUTHOR + YEAR	DESIGN	SETTING	INTERVENTION	COMPARATOR	N	PRIMARY OUTCOME	OTHER KEY OUTCOMES	ADVERSE EVENTS
**BLANCHER ET AL, 2019 (69)**	Double-blind multicenter non-inferiority RCT	ED	Sufentanil IN 0.30 µg/kg + placebo IV	Placebo IN + Morphine IV 0.1 mg/kg	136	Superior mean reduction in pain intensity as measured with NRS at 30 min: -5.2 (97.5%CI -5.7, -4.6) vs -4.1 (97.5%CI -4.6, -3.6), mean difference 1.1 (97.5%CI 0.3, 1.9), *p* < 0.001.	Similar mean pain reduction as measured with NRS at 10 and 20 min but values not reported.<br><br>Similar median patient satisfaction as measured on 100-point scale: 80 (IQR 70, 100) vs 80 (IQR 60, 92.5), *p* = 0.34.	Similar incidence of mild and severe AEs: 46.3% vs 60.9%, *p* = 0.09 and 9.0% vs 2.9%, *p* = 0.16. Severe AEs were hypoxemia, hypotension and bradypnea. Naloxone was not required.
**MALINVERNI ET AL, 2024 (70)**	Open-label single center RCT	ED	Sufentanil IN 0.5 µg/kg + SoC[1] excluding opiates	SoC[1]	170	Stronger median reduction in pain intensity as measured with VAS (0-10) at 15-20 min: 3.0 (IQR 1.7, 5.0) vs 1.5 (IQR 0.9, 3.0), *p* < 0.001.	Higher median pain reduction as measured with VAS (0-10) at 60 min: 5 (IQR 3, 7) vs 3 (2, 5.3), *p* < 0.001.<br><br>Similar percentage of patients required rescue medication: 24.1% vs 23%, *p* = 0.87)	Higher incidence of AEs: 71.1% vs 23%, *p* < 0.001. Most common were dizziness (54.2%), sweating (20.5%) and nausea (19.3%). Similar incidence of severe AEs: 7.2% vs 3.5%, *p* = 0.27.
**LEMOEL ET AL, 2019 (71)**	Double-blind single center RCT	ED	Sufentanil IN 0.4 µg/kg + SoC[2]	Placebo IN + SoC[2]	144	Higher proportion of patients experienced pain relief (NRS ≤3) at 30 min: 72.2% vs 51.4%, difference 20.8 (95%CI 4.0, 36.2), *p* = 0.01.	Lower proportion of patients received morphine titration: 31.9% vs 53.5%, difference 21.6% (95%CI 4.4, 37.2).<br><br>Similar mean patient satisfaction at discharge as measured with VAS (0-10 cm): 9.1 cm (SD 1.3) vs 9.3 cm (SD 0.3), difference -0.2 cm (95%CI -1.0, 0.7)	Higher incidence of opioid related AEs: 66.7% vs 22.5%, difference 44.1% (95%CI 27.2, 57.7). Most common were respiratory AEs (16.7% vs 2.8%), somnolence (22.2% vs 7.0%) and nausea and vomiting (33.3% vs 9.9%). Naloxone was not required. No SAEs.

**KREPS ET AL, 2023 (80)**	Open-label single center sequential period trial	ED	Sufentanil IN 0.7 µg/kg + SoC[1] excluding opiates	SoC[1]	138	Stronger median reduction in pain intensity as measured with VAS (0-10) at:<br><br>15 min: 2.5 (IQR 1.2, 4) vs 1.6 (1, 2.4), *p* = 0.005;<br><br>30 min: 4 (3, 5.7) vs 3.1 (2, 4.4), *p* = 0.02.<br><br>Similar reduction in pain intensity as measured with VAS at 60 min: 5 (IQR 4, 6) vs 4.3 (IQR 3, 5.8), *p* = 0.06.	Lower percentage of patients required IV access: 0% vs 6.4%, *p* = 0.015.<br><br>Higher percentage of patients received rescue medication beyond 15 min: 10.1% vs 4.3%, *p* = 0.018.	Higher incidence of AEs: 68.1% vs 0%. Most common were vertigo (60.4%), nausea (30.0%), vomiting (20.0%) and diaphoresis (20.0%).
**STEENBLIK ET AL, 2012 (81)**	Observational study	Ski clinic	Sufentanil IN 0.5 µg/kg	-	40	Mean reduction in pain intensity as measured with NRS was 4.7 (95%CI 3.7, 5.6) at 10 min, 5.8 (95%CI 4.8, 6.8) at 20 min and 5.7 (95%CI 4.7, 6.8) at 30 min.	Proportion of patients with inadequate pain control was 5%.<br><br>83% of nurses and 87% of physicians reported they were "very satisfied" with treatment.	Most common side effects were dizziness (7.5%), vomiting (2.5%) and hypoxia (2.5%).
**MINER ET AL, 2018 (67)**	Open-label multicenter feasibility trial	ED	Sufentanil sublingual tablet 30 µg	-	76	Mean pain intensity as measured with NRS was 8.1 at baseline, 7.0 after 15 min, 6.2 at 30 min and 5.2 at 60 min. NRS reduction was significant from baseline (*p* < 0.001).	7.5% of patients required rescue medication within 1 hour.<br><br>Administration was indicated as (somewhat) easy by 100% of hospital personnel when administered upright, by 83.4% when administered reclined, and by 85.7% when administered with limited lighting.	Incidence of treatment related AEs was 15%. Most common were nausea and vomiting (11%), somnolence (3%) and desaturation (3%). One SAE occurred: angina pectoris, moderate in severity.

95%CI = 95% confidence interval, AE = adverse event, cm = centimeter, ED = emergency department, IN = intranasal, min = minutes, NRS = numeric rating scale, RCT = randomized controlled trial, SAE = serious adverse event, SD = standard deviation SoC = standard of care, VAS = visual analogue scale.

[1]SoC consisted of paracetamol PO or IV 1 g, diclofenac PO 50 mg or ketorolac IV 20 mg and oxycodone PO 5 mg or titrated morphine IV.

[2]SoC consisted of paracetamol IV 1 g, ketoprofen IV 100 mg and titrated morphine IV (if NRS ≥6).
